# Effectiveness and safety of massage for athletic injuries: A protocol for systematic review and meta-analysis

**DOI:** 10.1097/MD.0000000000026925

**Published:** 2021-08-13

**Authors:** Guangxin Guo, Shengji Xie, Feihong Cai, Xu Zhou, Jianghan Xu, Boyi Wu, Guanghui Wu, Ran Xiao, Xiruo Xu, Ping Lu, Min Fang

**Affiliations:** a School of Acupuncture-moxibustion and Tuina, Shanghai University of Traditional Chinese Medicine, Shanghai 201203, China; b Yueyang Hospital of Integrated Traditional Chinese and Western Medicine, Shanghai University of Traditional Chinese Medicine, Shanghai 200437, China; c Research Institute of Tuina, Shanghai Academy of Traditional Chinese Medicine, Shanghai 200437, China.

**Keywords:** athletic injuries, massage, protocol, systematic review

## Abstract

**Background:**

Athletic injuries have been a major area of interest in the field of sports and clinical medicine. Implemented on people's skin, muscles, and joints as an important part of complementary and alternative medicine (CAM), massage therapy has a positive effect on athletic injuries. This protocol is to provide the methods used to evaluate the effectiveness and safety of massage therapy for patients with athletic injuries.

**Methods:**

A systematic search will be performed in the following electronic databases for randomized controlled trials (RCTs) to evaluate the effectiveness and safety of massage therapy in treating athletic injuries: PubMed, the Cochrane Library, EMBASE and four Chinese databases (CNKI, Wan Fang, CBMdisc and VIP). Each database will be searched from inception to July 2021. The entire process will include study selection, data extraction, risk of bias assessment and meta-analysis.

**Results:**

A high-quality synthesis of current evidence of massage therapy for patients with athletic injuries will be provided.

**Conclusions:**

This systematic review will provide evidence for assessing the credibility of massage therapy for patients with athletic injuries.

**Dissemination and ethics:**

The results of this review will be disseminated through peer-reviewed publication. This review does not require ethical approval because all the data used in this systematic review and meta-analysis have already been published. Furthermore, all of these data will be analyzed anonymously during the review process.

**INPLASY registration number:**

INPLASY202170066.

## 1 Introduction

Athletic injuries can be defined as the loss of physical function or structural integrity in athletic training or competition.^[1,2]^ With the increasing popularity of competitive sports worldwide, hot sports events have brought huge profits to athletes. A growing number of people are taking up sports and striving to become professional athletes, which aggravates the physical and mental burden of athletes, increases the required amount of training content and competitions, and places athletes who participate in the sport at a higher risk of injury.^[3–5]^ Such phenomenon has caused a significant economic burden.^[6]^ At present, athletic injuries have become a common health problem in the world. In Germany, 3.1% of German adults suffer from athletic injuries in a year, which are the second most common type of accidents after domestic accidents, and for those sport population, the annual injury probability reaches 5.6%.^[7]^ Therefore, it is very important to find effective and safe treatment methods to improve the recovery speed of sports participants and reduce their medical burden.

Currently, the main treatment modalities for athletic injuries are medications and CAM treatments. In order to reduce or prevent the symptoms of athletic injuries, many people use traditional physical therapy strategies, such as massage, cold water immersion and so on.^[8–10]^ A survey of intercollegiate student athletes showed that 56% of the subjects used CAM within one year, and massage, as the most commonly used type of treatment, accounted for 38%.^[11]^ However, scientific evidence is still not enough to prove the effectiveness of massage in the treatment of athletic injuries, and it is still an issue worthy of exploration.

Massage therapy, one of the most effective and widely used CAM,^[12]^ is also one of the most common techniques used by athletes to recover and enhance their athletic performance after exercise. Under the operation of well-trained professionals, massage is considered to be a safe and effective treatment without any significant risks or side effects. At present, scientific and technological research in this field is developing rapidly. Studies have shown that the benefits of massage therapy include reduced post-exercise pain and perceived fatigue,^[13,14]^ attenuated inflammatory signals of exercise-induced muscle injury,^[15]^ improved muscle strength and self-perception of exercise-induced muscle injury,^[16,17]^ restoration of heart rate variability and diastolic blood pressure,^[18]^ acute relief of muscle soreness after exercise,^[19]^ reduced muscle tension, post-exercise serum creatine kinase, swelling and respiratory pattern disorders, which help to prevent injuries.^[20–22]^

Meta-analysis is a powerful statistical technique that is widely accepted as an important tool for evidence-based medicine.^[23]^ So far, there is no meta-analysis article reporting on the effectiveness and safety of massage for athletic injuries. Therefore, we perform this protocol to comprehensively assess the effect of massage on athletic injuries. Despite the growing popularity of massage therapy, the discussion about its effectiveness in CAM continues. Compared with other therapies, massage therapy has the advantages of non-invasive and relatively low cost. Therefore, it is necessary to pay attention to the research and development in this field.

## 2 Methods

### 2.1 Study registration

This protocol was registered on the International Platform of Registered Systematic Review and Meta-analysis Protocols (INPLASY) on July 21, 2021 (registration number: INPLASY202170066). We will strictly perform this protocol by following the Preferred Reporting Items for Systematic Reviews and Meta-analysis Protocol (PRISMA-P) statement guidelines.^[24]^

### 2.2 Inclusion criteria for study selection

#### 2.2.1 Type of studies

Only RCTs about massage for athletic injuries will be included, with language restrictions in English or Chinese. Case report, experience report, and laboratory studies will not be included.

#### 2.2.2 Types of participants

All patients with athletic injuries will be included without limitation of age, race, gender, economic level, and severity.

#### 2.2.3 Types of interventions

##### 2.2.3.1 Experimental interventions

The interventions of experimental group will only consist of massage therapies, mainly including general massage, acupressure, Chinese massage, relaxation, manual lymphatic drainage and so on. There will be no limitation on the methods, duration, and frequency of massage.

##### 2.2.3.2 Control interventions

The interventions of control group will involve any therapy other than massage (e.g., medication, placebo, routine care, etc.).

#### 2.2.4 Types of outcome measures

##### 2.2.4.1 Primary outcomes

Visual analogue scale (VAS).

##### 2.2.4.2 Additional outcomes

1. Frequency of delayed onset of muscle soreness.
2. A 12-item Short-Form Health Survey (SF-12).
3. Pressure pain threshold.
4. Numerical rating scale of soreness intensity.
5. Adverse events.

### 2.3 Search strategy

We will perform a comprehensive search in PubMed, the Cochrane Library, EMBASE and four Chinese databases (CNKI, Wan Fang, CBMdisc, and VIP) for articles published before July 2021. Only RCTs that used massage as the main treatment for adults with athletic injuries will be included. The Chinese and English search strategies in PubMed database are shown in Table 1. The search terms in the Chinese databases have the same meaning as those used in the English databases. There will be no language restrictions in this review.

**Table 1:** Search terms used in Pubmed database.

Search strategy
#1 Intervention: ((((((“Massage Therapy”) OR “Massage Therapies”) OR “Therapies, Massage”) OR “Therapy, Massage” OR “Tuina” OR “Manipulation” OR “Manual therapy”)) AND “Massage”[Mesh]
#2 Participant: (“Athletic Injuries”[Mesh]) AND ((((((((((((((((((Injuries, Sports) OR Injury, Sports) OR Sports Injury) OR Sports Injuries) OR Injuries, Athletic) OR Athletic Injury) OR Injury, Athletic) OR Athletic Performance) OR Sports Performance) OR Performance, Sports) OR Exercise-induced Muscle Damage) OR EIMD) OR Sprains) OR Strains) OR torn ligaments) OR ligaments, torn) OR ligaments, rupture) OR ligaments, flabby))
#3 Study desgin: (randomized controlled trial [pt] OR controlled clinical trial [pt] OR randomized [tiab] OR placebo [tiab] OR clinical trials as topic [mesh: noexp] OR randomly [tiab] OR trial [ti])NOT (animals [mh] NOT humans [mh])
#4 #1 AND #2 AND #3

### 2.4 Identification of studies

**Figure 1:**
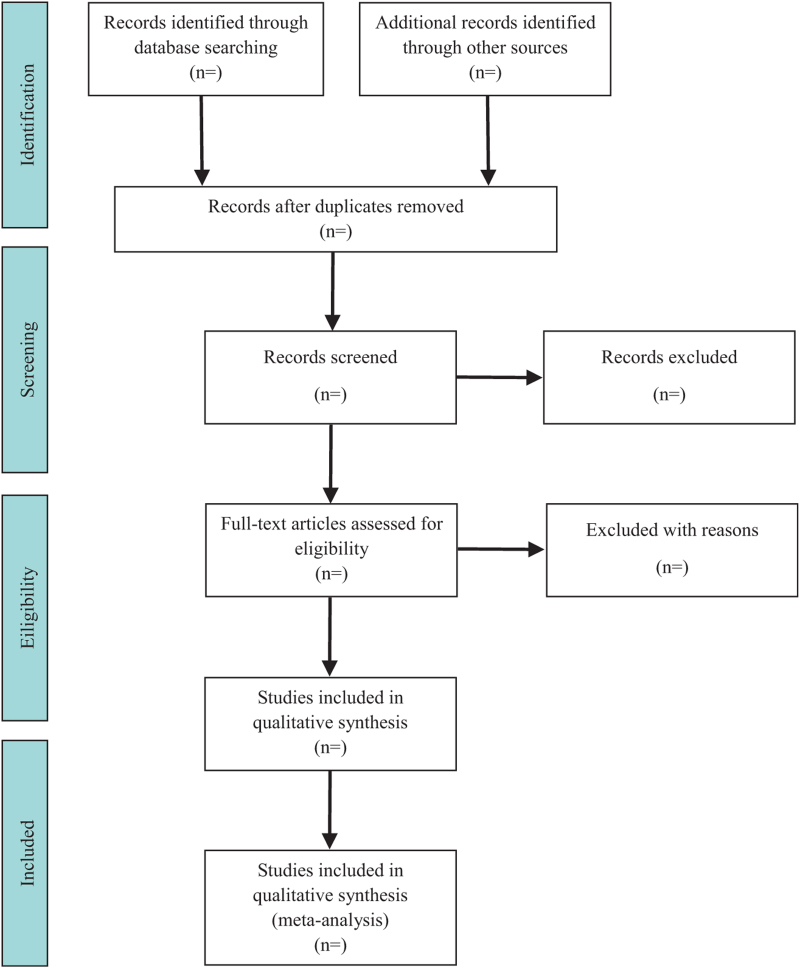
Flow diagram of study selection which is composed of four parts. First, we need to search the literature in both domestic and international databases. The second step is the screening of the title and abstract of the articles. The third step is choosing the eligible studies. The last step is including the studies which is in qualitative synthesis.

All the search results will be imported into EndNote software (V.x9) for management. Two reviewers (SX and FC) will independently screen all potentially eligible studies. Titles and abstracts will be screened first to exclude irrelevant citations. Full text of all the articles with potentially relevant abstracts will be retrieved and screened according to the study eligibility criteria. Disagreements will be resolved by consensus or discussion with a third reviewer (GG). The research flow chart is shown in Figure 1.

### 2.5 Data collection

Two reviewers will extract data from the included literature through Microsoft Excel 2010 (Microsoft company, Seattle, USA), mainly including the following information (Table 2):

(1) General information about the study, such as authors, year of publication, country, groups, sample size, age and gender;
(2) Detailed treatment information, such as diagnostic criteria and parameters of intervention;
(3) Pain scores. Other outcome measurements, such as SF-12 or Frequency of delayed onset of muscle soreness, will be extracted if they are mentioned in the study.

**Table 2:** Data extraction form.

First authors	Year	Country	Sample size	Mean age
Gender	Pain location	Duration	Follow-up	Diagnostic criteria
Experimental group intervention	Control group intervention	Main outcome assessments		

### 2.6 Quality of evidence assessment

Based on Grading of Recommendations Assessment Development and Evaluation, we will assess the quality of evidence as 4 grades: high quality, moderate quality, low quality and very low quality.^[25]^ In addition, we will use the online guideline development tool to conduct this process.

### 2.7 Risk of bias assessment

Study quality will be assessed in RevMan V5.4 (the Nordic Cochrane Centre, Cochrane Collaboration)^[26]^ using the Cochrane risk of bias tool. The risk of bias for each of the following domains will be assessed for each study:

1. random sequence generation,
2. allocation concealment,
3. blinding of participants and personnel,
4. blinding of outcome assessments,
5. incomplete outcome data,
6. selective reporting, and
7. other bias.

Each study included will be rated as having a high, low, or unclear risk of bias. Two reviewers (SX and FC) will evaluate the consistency of all the extracted data and quality ratings. Disagreements will be resolved by discussion with a third reviewer (GG).

### 2.8 Statistical analysis

Revman 5.4 software will be used to perform statistical analysis. For discontinuous variables, the risk ratio (RR) with 95% confidence interval (CI) will be selected. For continuous variables, the weighted mean difference (WMD) with 95% CI will be selected when the measuring instruments are the same, and the standardized mean difference (SMD) with 95% CI will be selected when the measuring instruments are different. We will use the fixed-effect model if there is no significant heterogeneity (*P* > .1 or *I*^*2*^ < 50%). If there is a significant heterogeneity (*P* > .1 or *I*^*2*^ < 50%), we will conduct subgroup analysis or sensitivity analysis to identify possible causes of heterogeneity among populations.

### 2.9 Subgroup analysis

If the necessary data are available, subgroup analysis will be conducted according to the following criteria:^[27]^

(1) The treatment period.
(2) Different acupuncture points with massage.
(3) Different types of manipulation (e.g., kneading, rolling, pressing).

### 2.10 Sensitivity analysis

To identify the robustness of the meta-analysis, low-quality trials, with high risks of bias or outcomes that are seriously distant from the rest of the data, will be excluded.

### 2.11 Ethics and dissemination

Ethical approval will not be in need because the data used in this systematic review will not be individual patient data, and there will be no concerns regarding privacy.

## 3 Discussion

Athletic injuries are experienced by most athletes on an annual basis. Researchers have found that injuries are one of the most common reasons for stopping sports.^[28]^ Proper exercise, use of protective equipment, and maintaining bone health can effectively prevent athletic injuries.^[29]^ Acupuncture and massage, as therapies with fewer side effects, have also achieved excellent results in the treatment of athletic injuries.^[30,31]^ High-frequency massage can alter the expression of key transcription factors that regulate inflammation (such as NF-кβ). In addition, massage leads to an increase in mitochondrial biogenesis and a decrease in heat shock protein 27 phosphorylation, thereby reducing cellular stress caused by muscle fiber injury.^[32]^ Although many studies have reported the effectiveness of these supplementary interventions, there is a general lack of determination of the most effective therapeutic massage methods in the field of athletic injuries. Therefore, this online meta-analysis will provide a detailed summary and analysis of the latest evidence, with a focus on available massage methods. We hope that our findings will help patients, clinicians, and healthcare policy makers make better treatment choices for athletic injuries.

## Author contributions

**Funding acquisition:** Guangxin Guo, Ping Lu, Min Fang, Jianghan Xu.

**Methodology:** Guangxin Guo, Shengji Xie, Ping Lu.

**Project administration:** Guangxin Guo, Ping Lu, Min Fang.

**Supervision:** Guangxin Guo, Shengji Xie.

**Writing – original draft:** Guangxin Guo, Shengji Xie, Feihong Cai, Xu Zhou, Jianghan Xu, Boyi Wu, Guanghui Wu, Ran Xiao, Xiruo Xu, Ping Lu.

**Writing – review & editing:** Guangxin Guo, Shengji Xie, Feihong Cai, Xu Zhou, Jianghan Xu, Boyi Wu, Guanghui Wu, Ran Xiao, Xiruo Xu, Ping Lu.
